# Supplementary material for: Two-Dimensional Indium Selenide for Sulphur Vapour Sensing Applications
Source: Nanomaterials (Basel). 2020 Jul 18;10(7):1396. doi: 10.3390/nano10071396 (PMC7408355; doi:10.3390/nano10071396)
Supplement: Supplementary file 1 [file nanomaterials-10-01396-s001.pdf]

nanomaterials

SI

Two-Dimensional Indium Selenide for Sulphur Vapour Sensing Applications

Daniel Andres-Penares [1,†], Rodolfo Canet-Albiach [1], Jaume Noguera-Gomez [1], Juan P. Martínez-Pastor [1,2], Rafael Abargues [1] and Juan F. Sánchez-Royo [1,2,*]

1 ICMUV, Instituto de Ciencia de Materiales, Universidad de Valencia, P.O. Box 22085, 46071 Valencia, Spain; Daniel.Andres@uv.es (D.A.-P.); Rodolfo.Canet@uv.es (R.C.-A.); Jaume.Noguera@uv.es (J.N.-G.); Juan.Mtnez.Pastor@uv.es (J.P.M.P.); rafael.abargues@uv.es (R.A.)

2 MATINÉE: CSIC Associated Unit-(ICMM-ICMUV of the University of Valencia), Universidad de Valencia, P.O. Box 22085, 46071 Valencia, Spain

* Correspondence: Juan.F.Sanchez@uv.es

† Current address: Institute of Photonics and Quantum Sciences, SUPA, Heriot-Watt University, Edinburgh EH14 4AS, UK.

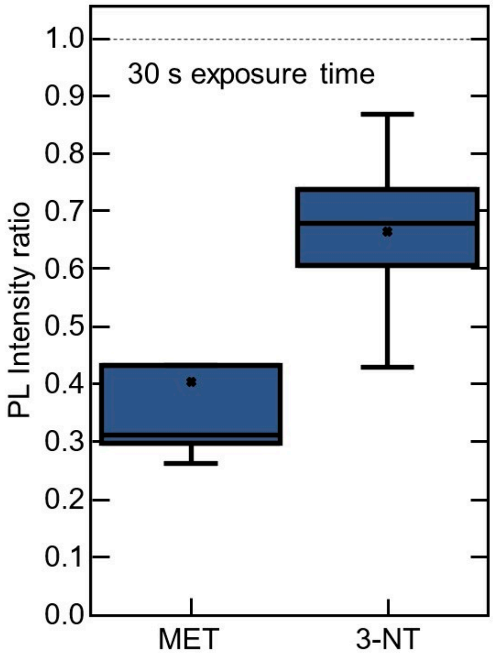

Figure S1. Vapour sensing with InSe. Integrated PL intensity measured in 2D InSe nanosheets after 30 s of exposure to MET 99 % and 3-NT 99 %. The integrated PL signal has been normalized to that measured in each sample before vapour exposure. For each vapour, error bars, mean value (marked as an ×), median (horizontal line), and quartile calculation using inclusive median method (dark-blue rectangle) have been obtained using all the statistics collected for that specific exposure vapour.
